# Outcomes of Differentiated Thyroid Cancer in Children and Adolescents at King Abdulaziz Medical City, Jeddah

**DOI:** 10.7759/cureus.65992

**Published:** 2024-08-02

**Authors:** Tariq Nasser, Bsaim Abdulsalam Altirkistani, Suaad Muhammad Bougis, Asma Hassan Abu Ghasham, Ibrahim Basem Nafadi

**Affiliations:** 1 College of Medicine, King Saud bin Abdulaziz University for Health Sciences, Jeddah, SAU; 2 Department of Medicine/Endocrinology, King Abdullah International Medical Research Center, Jeddah, SAU; 3 Department of Medicine/Endocrinology, King Abdulaziz Medical City, Jeddah, SAU

**Keywords:** thyroid nodule malignancy, pediatric thyroid cancer, radioactive iodine (rai) therapy, follicular thyroid carcinoma, papillary thyroid carcinoma, differentiated thyroid cancer

## Abstract

Objective

Differentiated thyroid cancer (DTC) is rare in the pediatric population, with most data from the Western world. We aimed to describe the clinical presentation, treatment intervention, histopathological characteristics, complications, follow-up, and response to treatment in 17 patients with DTC at or below the age of 20 years.

Interventions

This was a retrospective cohort study at King Abdulaziz Medical City, Jeddah, Saudi Arabia. We included patients aged younger than 20 years with DTC. Total or near-total thyroidectomy was performed in 82% of the patients, central and/or lateral neck dissection in 35% of cases, and radioactive iodine (RAI) ablation in 76% of cases.

Results

The study included 17 patients (14 females), with a median age of 16 years at the time of diagnosis. Thyroid nodules were the main complaint in 88% of the patients. Thyroid ultrasonography was the main method for the initial evaluation. Papillary cancer was the most common type of tumor, and lymph node spread was found in 82% of the patients. Moreover, 40% of the patients exhibited excellent responses to therapy, with 35% showing indeterminate results. Only 23.5% of the patients developed hypocalcemia postoperatively.

Conclusions

Classical papillary thyroid carcinoma was the predominant histopathological type, and most patients showed excellent responses to therapy, followed by indeterminate in most of the cases. The most common presentation was a neck nodule, signifying the role of thorough physical neck examinations. Finally, recurrence occurred in a minority of patients. However, none of these patients died.

## Introduction

Although endocrine malignancy is uncommon among pediatric patients, it represents approximately 6% of all pediatric cancers [1]. The incidence of thyroid cancer (TC) has been increasing worldwide, which may be explained by factors such as the increased sensitivity of the detection of small carcinomas, the deficiency of iodine, head and neck radiation exposure, and family history [2-4]. Furthermore, papillary and follicular carcinomas, the most common histopathological types of thyroid tumors, are considered well-differentiated cancers that resemble normal thyroid tissues, causing difficulty in distinguishing between normal and cancerous tissues [5]. In the pediatric group, most patients present with papillary carcinoma in approximately 80%-90% of cases, the remaining 10% present with a follicular type, while only 3%-5% have medullary cancer [1]. In Saudi Arabia, thyroid cancer is the second most common cancer among adult Saudi females and the eighth most common cancer among Saudi males, with a female-to-male ratio of 3:1. The incidence of thyroid cancer in pediatric patients in Saudi Arabia is 0.1-0.5 per 100,000 persons [6]. Patients commonly present with thyroid nodules that can manifest as asymptomatic neck masses, as well as compressive and functional symptoms such as dyspnea and signs of hyperthyroidism [1]. In diagnostic assessments, fine-needle aspiration (FNA) is considered the cornerstone for determining the malignancy of thyroid nodules [1].

Total thyroidectomy is the treatment of choice for most cases of thyroid cancer. It is usually performed along with postoperative radioiodine ablation and thyroid hormone suppression to eradicate any remaining viable thyroid tissue [7]. The long-term outcomes of differentiated thyroid cancer (DTC) in pediatric patients have shown favorable prognosis and responsiveness to treatment, with a 10-year survival rate as high as 98.8% [8]. However, patients younger than 20 years are often more likely to present with aggressive, advanced-stage disease. As the disease progresses, they also show a higher rate (53%) of developing lymph node metastasis. Lung metastasis is another possible presentation with rates ranging from 7% to 30% and variations in the recurrence rate after thyroidectomy [8-12]. The outcomes, including the complete remission, recurrence, or persistence of tumors after therapy, can be determined and evaluated by measuring serum thyroglobulin (Tg) levels and performing neck ultrasonography and diagnostic radioiodine whole-body scanning (DxWBS) [10].

Studies on thyroid cancer in children, specifically in Saudi Arabia, are limited because of the infrequency of the disease and its unusual course in pediatric patients. Therefore, this study focused on assessing the various clinical presentations, prognoses, and outcomes of differentiated thyroid cancer in the pediatric population at our tertiary care center.

This article was accepted for poster presentation at the American Association of Clinical Endocrinology (AACE) Communities Middle East and North Africa (MENA) Conference, which was held in Dubai, United Arab Emirates (UAE), from November 17 to 19, 2022.

## Materials and methods

Study settings and design

A record-based retrospective study with a cohort design was conducted in King Abdulaziz Medical City, Jeddah, Saudi Arabia, from December 2010 to December 2021. The exclusion criteria include patients with undifferentiated thyroid cancer in the histopathological report and patients more than the age of 20 years old at the time of diagnosis.

The study was approved by the Institutional Review Board of King Abdullah Medical City (KAMC) (approval number: NRJ21J/197/08). Informed consent was waived due to the retrospective nature of the study.

Identification of study participants

Patients diagnosed with well-differentiated thyroid cancer and aged younger than or equal to 20 years at diagnosis were included. Patients with undifferentiated thyroid cancer were excluded from this study.

Data collection process

Data were obtained from the hospital's electronic records by using BESTCare 2.0 (ezCaretech, Torrance, CA). We used a data collection form that assessed the study variables, including demographic data such as age, sex, family history of endocrine disorders, and history of radiation. Additionally, the clinical presentation, response status, and management plan were retrieved and obtained from the admission notes. The surgical section notes were assessed to determine whether total or subtotal thyroidectomy was performed and if complications occurred. The pathology report was evaluated to determine the type of cancer, lymph node involvement, and the presence of metastasis. Finally, radiology and laboratory results were obtained. Then, these data were retrieved, collected, and entered manually on an Excel sheet (Microsoft Corp., Redmond, WA) by the research team and kept confidential.

Data analysis

Data analysis was performed using the JMP software (JMP Statistical Discovery LLC, Cary, NC). Nonparametric measures of the central tendency and variance were used. Continuous variables were reported as median and interquartile ranges (IQRs), and categorical variables were reported as frequencies and percentages. Fisher's exact test was used for categorical analysis. Statistical significance was set at p < 0.05.

## Results

Seventeen patients met the inclusion criteria for this study. Of these, 14 (82%) were female. The median age at diagnosis was 16 years (IQR: four years). A family history of endocrine-related diseases was found in three (18%) patients, but none of the patients reported endocrine-related malignancies. Only one patient had a history of radiation exposure. Thyroid nodules were the most common clinical manifestation in 14 patients (88%). All patients were initially evaluated using ultrasound, but none underwent genetic testing. The tumor site predominantly involved the right thyroid in 11 patients (69%), with a median size of 2.85 cm (IQR: 2.82 cm). The final histopathology report revealed that the classical type of papillary thyroid cancer was the most frequently reported form (11 patients, 66%), followed by the follicular variant (four patients, 23%) and the non-invasive follicular thyroid neoplasm variant (one patient). In contrast, follicular carcinoma was found in only one patient who had the minimally invasive type.

Moreover, lymph node metastasis was reported in 14 patients (82%). Only one patient had distant metastasis in the lung. More details are provided in Table 1.

**Table 1:** Demographic characteristics and additional information

	N (%)
Sex	
Male	3 (18)
Female	14 (82)
Age, median (interquartile range)	16 (4)
Family history of endocrine disorder	3 (18)
History of radiation	1 (6)
Clinical manifestations	
Thyroid nodule	14 (88)
Neck pain	2 (13)
Fatigue	2 (13)
Palpitation	2 (13)
Shortness of breath	1 (6)
Sweating	1 (6)
Muscle ache	1 (6)
Fever	1 (6)
Papillary carcinoma	
Classic	11 (65)
Follicular variant	4 (23)
Non-invasive follicular thyroid neoplasm with papillary-like nuclear features (NIFTP)	1 (6)
Follicular carcinoma	
Minimally invasive follicular carcinoma	1 (6)
Lymph node spread	14 (82)
Tumor site	
Right	11 (69)
Isthmus	7 (44)
Left	7 (44)
Tumor size, median (interquartile range)	
Right	2.85 (2.82)
Isthmus	2.25 (1.55)
Left	1.3 (1.7)

Most of our patients underwent total thyroidectomy (n = 14, 82%), whereas only three patients (18%) were treated with initial subtotal thyroidectomy; they underwent a second surgical procedure after a review of the final pathology result of the initial procedure or in the follow-up. Furthermore, nodal dissection was performed in seven (41%) patients, based on tumor size and preoperative ultrasound and CT findings, of whom five (71.4%) underwent central neck dissection. Post surgery, it is imperative to note that four patients experienced postoperative hypocalcemia, with calcium levels falling below 2.1 mmol/L. One of these patients developed permanent hypocalcemia. This emphasizes the critical importance of effectively monitoring and managing postoperative care to ensure optimal outcomes. In contrast, permanent hypocalcemia that required calcium and vitamin D replacement therapy was seen in one patient (5.9%). Thirteen (76.0%) patients underwent therapeutic radioactive iodine (RAI), ranging from one to three doses. The first RAI treatment dose was given 18 ± six weeks postoperatively; eight received the first dose of RAI within 12 weeks after surgery. Suppressive thyroid hormone therapy was applied in all patients. Before the surgery, the median thyroid-stimulating hormone (TSH) level was 1.68 (1.59). In addition, the median first measurements of Tg were 8.85 (0.2-70 ng/mL). More data regarding patient management and postoperative complications are provided in Table 2.

**Table 2:** Management and postoperative complications TSH, thyroid-stimulating hormone; Tg, thyroglobulin

	N (%)
Management	
Total thyroidectomy	14 (82)
Subtotal thyroidectomy	3 (18)
Nodal dissection	6 (35)
Central neck dissection	5 (83)
Cervical neck dissection	1 (17)
Lateral neck dissection	1 (17)
Complications post surgery	
Hypocalcemia (temporary)	3 (18)
Hypocalcemia (permanent)	1 (6)
Fever	2 (12)
Temporary hoarseness of voice	3 (18)
Permanent voice change	0
TSH (0.5-4.8 mIU/L) before surgery, median (interquartile range)	1.68 (1.59)
First postoperative Tg (0.2-70 ng/mL) measurement, median (interquartile range)	8.85 (93.97)

These patients were scheduled to be followed up every six months for clinical examination, serum TSH levels, thyroglobulin, T4, chest X-ray, and neck ultrasound. The mean duration of follow-up was 82.8 months (range: 18-129 months). One year after the initial surgery and at each follow-up visit, response to treatment was measured using the American Thyroid Association (ATA) response to therapy systems (see Table 3).

**Table 3:** ATA response to therapy systems Definitions, clinical outcomes, and management implications of the ATA response to therapy categories [13] US, ultrasound; Tg, thyroglobulin; stim, stimulated; Ab, antibody; TSH, thyroid-stimulating hormone; ATA, American Thyroid Association

Response to therapy	Recommendation
Excellent response: negative US-suppressed Tg <0.2 or stim <1.0	Decrease intensity and frequency of follow-up and TSH suppression
Incomplete biochemical response: negative US-suppressed Tg <1.0 or stim <10.0 and rising Tg Ab	Stable/lower Tg observed and rising Tg and additional investigations and therapies
Incomplete structural response: structural evidence of disease	Consider additional investigations and therapies
Indeterminate response: Tg Ab+ stable or declining suppressed Tg <0.2-1.0 or stim <1.0-10	Continued observation and testing

Most patients showed excellent and indeterminate responses, which were observed in seven (41%) and six (35%) patients, respectively; however, two patients (12%) showed a biochemically incomplete response initially with suppressed Tg of 0.4 and 0.7, which declined to <0.2 within three years. Two (12%) showed a structurally incomplete response, which required additional doses of 131I. Lastly, recurrence occurred in two patients (12%) who had received additional therapy with radioactive 131I. However, there was no complete data regarding the doses. Nevertheless, our survival rate was 100%, but the two patients who had recurrence still had residual disease. More details are presented in Table 4 and Table 5.

**Table 4:** Outcomes of differentiated thyroid cancer

	N (%)
Response status	
Excellent response	7 (41)
Indeterminate response	6 (35)
Biochemical incomplete response	2 (12)
Structural incomplete response	2 (12)
Risk of tumor	
Low risk	4 (24)
Intermediate risk	7 (41)
High risk	6 (35)
Recurrence	2 (12)

**Table 5:** Response status based on the type of cancer

Type of cancer	Response status	N (%)
Classic	Excellent response	4 (36.36)
	Indeterminate response	5 (45.45)
	Structurally incomplete response	2 (18.18)
Minimally invasive follicular carcinoma	Excellent response	1 (100)
Non-invasive follicular thyroid neoplasm with papillary-like nuclear features (NIFTP)	Excellent response	1 (100)
Papillary thyroid cancer with follicular variant	Biochemically incomplete response	2 (50)
	Excellent response	1 (25)
	Indeterminate response	1 (25)

## Discussion

Differentiated thyroid carcinoma in children has a higher risk of being extensive and aggressive with evidence of local or distant metastasis than in adults [14]. They also have a higher risk of recurrence than adults with thyroid carcinoma [1]. Although differentiated thyroid cancer in both adult and pediatric populations was managed with similar treatment methods, the pediatric population showed better outcomes [8]. Like the findings reported in other studies, thyroid cancer in our population predominated among females (82%) [2,3]. Even though a family history of thyroid cancer is a well-known risk factor for thyroid cancer, none of our patients reported a family history of thyroid cancer. Asymptomatic thyroid nodules were the most common presentation among our patients, which reflects the importance of neck examination, especially because of the higher potential of neck mass malignancy in pediatric patients than in adults [14].

Differentiated thyroid cancer, specifically the papillary and follicular types, is the most common thyroid malignancy, and its incidence has been increasing in both adult and pediatric populations [2]. Although lymph node involvement, which was frequent among our patients, is a predictor of a more aggressive tumor and a higher mortality rate, none of our patients died [10]. The ATA task force further recommends adjuvant radioactive iodine (RAI) for unresectable iodine-avid persistent locoregional disease (due to the invasion of vital structures) and/or distant metastases, which may be further guided by postoperative thyroglobulin levels [15]. In our institution, most patients were treated with a total thyroidectomy procedure (82%). Although this treatment, along with postoperative radioiodine therapy, is currently recommended for the management of pediatric thyroid cancer, many authors have questioned the aggressiveness of this treatment approach, given the long lifespan of these patients and the long-term complications of high doses of radioiodine [16]. Biological and clinical hypocalcemia are the most common complications of thyroidectomy [8]. Interestingly, only 21% of the patients in our study had hypocalcemia, and the remaining patients had normal to borderline normal calcium levels postoperatively. Despite the good responses frequently reported after managing pediatric thyroid malignancies [8], only 41% of our patients had an excellent response. In contrast, 59% had indeterminate or biochemically or structurally incomplete responses.

This study has a few limitations. First, the study retrospectively used patient charts, which may have compromised the quality and validity of the data. Second, the rarity of thyroid malignancies among children led to a small sample size, which limited the ability to compare and draw conclusions. Third, considering the slow course of TC, this is a relatively short time of median follow-up (6.9 years). In addition, the data for some aspects were missing for a few patients, which added to the difficulty in data analysis. However, the findings represent a valid addition to the limited number of reviews on pediatric thyroid malignancies, and we recommend future prospective, multicenter studies with larger study populations to improve treatment outcomes and reduce morbidity.

## Conclusions

In conclusion, we found that papillary thyroid carcinoma was predominant in our patients. Further, indeterminate and excellent responses were the most frequently reported. Although recurrence was reported in two patients, no mortality was observed in the study population. Our study emphasizes the importance of proper neck examination for early thyroid carcinoma detection since most patients presented with only neck nodules. Constant monitoring with closure follow-up and proper transfer care from pediatric to adult endocrinology are needed to recognize and treat promptly any local or distant metastasis. A temporal extension of the study will allow more reliable conclusions.
